# Breast metastasis of primary colon cancer with micrometastasis in the axillary sentinel node: A metastasis that metastasized?

**DOI:** 10.1186/1746-1596-6-45

**Published:** 2011-05-28

**Authors:** Tiziana Perin, Vincenzo Canzonieri, Lorenzo Memeo, Samuele Massarut

**Affiliations:** 1 Division of Pathology, CRO - Centro di Riferimento Oncologico, Istituto Nazionale Tumori, Via Franco Gallini, 2 - 33081 Aviano (PN) Italy; 2 Division of Breast Surgical Oncology, CRO - Centro di Riferimento Oncologico, Istituto Nazionale Tumori, Via Franco Gallini, 2 - 33081 Aviano (PN) Italy; 3 Pathology Unit, Department of Experimental Oncology, Mediterranean Institute of Oncology, Via Penninazzo,7 - 95029 Viagrande, (CT), Italy

## Abstract

A case of single breast metastasis from colon adenocarcinoma, with omolateral axillary micrometastasis, is reported with a brief review of the pertinent literature. The originality of the oncological concept of metastasis from metastasis, through lymphatics penetration, is discussed in the setting of a rare condition of breast metastasis from a colorectal carcinoma.

The demonstration of axillary lymph node micrometastasis has been possible because fine needle aspiration cytology of the breast nodule was suspicious, but not conclusive for metastasis from colon cancer, so lumpectomy with sentinel node biopsy was planned.

Although no disseminated nodal metastases were evident on computerized tomography scan and ultrasonography before breast surgery, the patient developed brain metastases and deteriorated rapidly; she died 16 months after presenting with the breast mass.

In conclusion, solid cancers are able to further metastasize, via well-known pathways also recognized in primary cancers such as neoplastic cell invasion of peritumoral lymphatics.

## Background

Metastasis to the breast from colon adenocarcinoma is very rare. Prognosis is poor because it is usually indicative of disseminated disease. It is important to distinguish metastatic disease from primary breast carcinoma in order to better planning the appropriate treatment [1].

The three main routes of tumour metastasis are direct spread, via the lymphatic system, and blood borne. It is however a matter of debate whether metastatic deposits have an ability to produce further metastases. In literature there are very few anecdotal data and experimental models have been inconclusive [2].

This case report describes a patient with breast metastasis from colon adenocarcinoma treated by lumpectomy and sentinel node biopsy. Strangely enough the sentinel node showed a micrometastasis from colon cancer. A possible interpretation of this finding might imply that distant metastases are able to further metastasize.

## Case presentation

In May 2005 a 46 year old woman underwent a sigmoid resection for colon cancer pT3 N1 M0 G2. Then she received 6 cycles of chemotherapy (FOLFOX). She remained well till April 2007 when she developed bilateral lung metastases treated with FOLFIRI and Avastin^® ^(Bevacizumab) with a partial prolonged response.

In May 2008 she was referred to the Breast Unit of Centro di Riferimento Oncologico (CRO) for a 1 cm palpable lump in her lower-outer aspect of the right breast.

Mammography was negative whereas ultrasounds showed a 1 cm nodule with irregular margins. Axillary lymph nodes were normal. Fine needle aspiration cytology (FNAC) was suspicious, but not conclusive for metastasis from colon cancer. Clinical diagnosis was: malignant right breast lump consistent either with metastasis from colon cancer or, albeit less likely, with a primary breast cancer. After discussing with the patient, we decided to perform a lumpectomy and a sentinel lymph node (SNL) biopsy. In the follow-up, the patient had a worsening of her disease, and she died of disseminated disease 16 months after her breast surgery.

**Figure 1:**
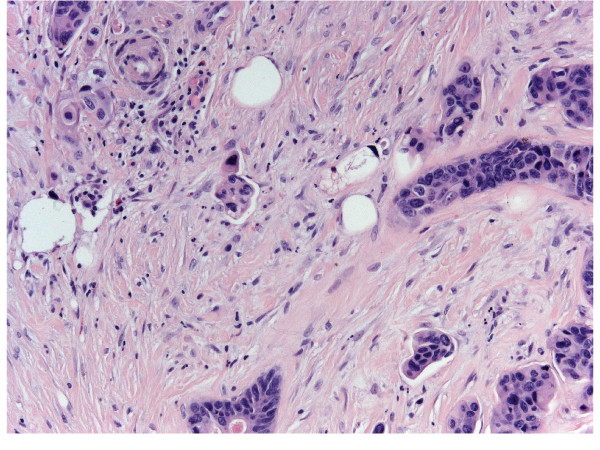
**Breast metastasis from colon cancer**. H&E Original magnification 25x.

Gross examination revealed a 1 × 1 cm hard lump in the lower outer quadrant of the right breast which was not fixed to the skin or underlying structures. Histologically the lesion showed irregularly shaped tubules and branching glands lined by cuboidal epithelial cells that exhibited pleomorphism and polarization (Figure 1). Lymph-vascular invasion (LVI) was detected at the periphery of the nodule. SLN was examined completely in serial sections carried out on paraffin embedded tissues as previously reported [3].

**Figure 2:**
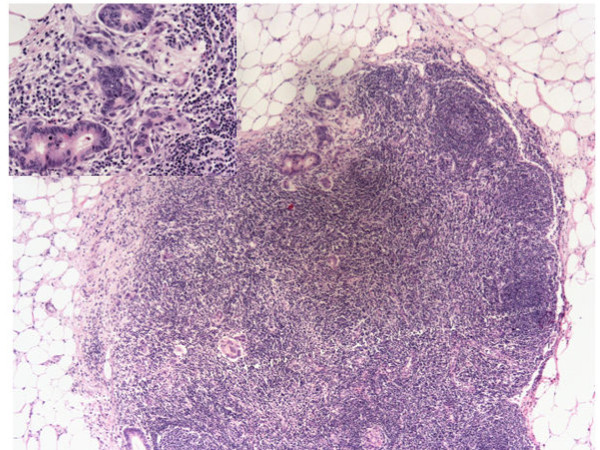
**SNL micrometastasis possibly derived from breast metastasis from colon cancer**. Original Magnification 10x. Insert: Cancer cells. Original Magnification 25x.

**Figure 3:**
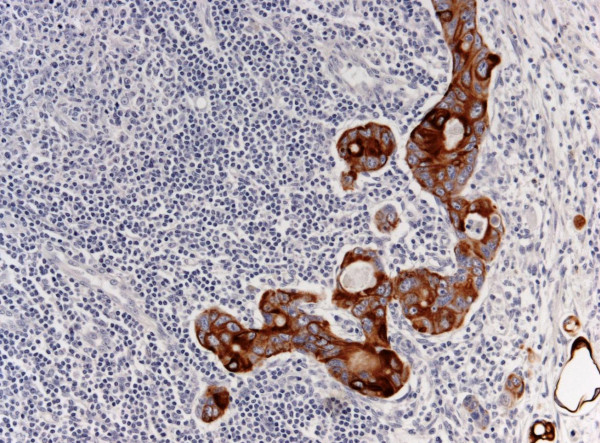
**SNL cancer cells positive for CK20**. Original Magnification 25x. H&E counterstain.

**Figure 4:**
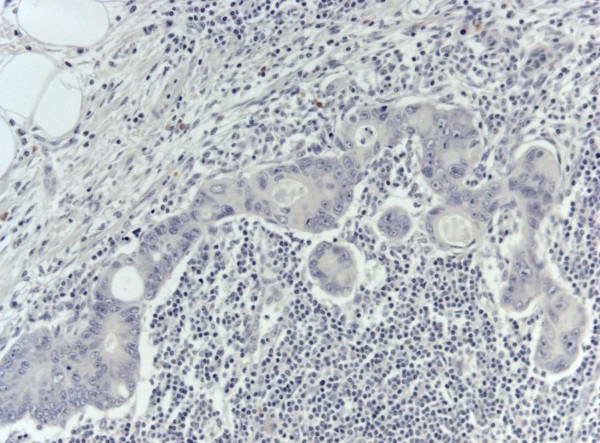
**SNL cancer cells negative for CK7.** Original Magnification 25x. H&E counterstain.

**Figure 5:**
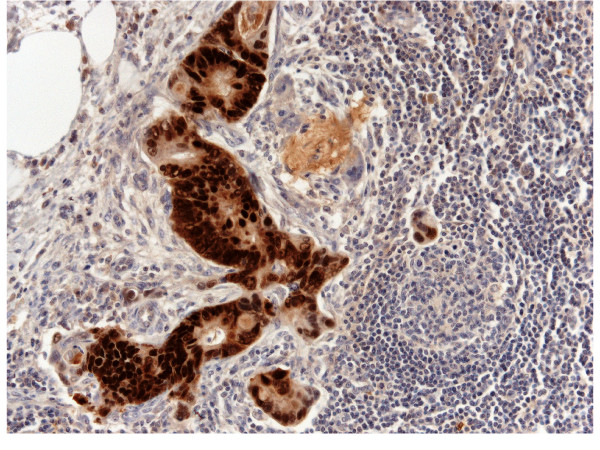
**SNL cancer cells positive for CDX2.** Original Magnification 25x. H&E counterstain.

A micrometastasis was found in SNL (Figure 2). Immunohistochemistry was performed both on breast lesion and sentinel biopsy metastasis: the neoplastic cells were negative for estrogen and progesterone receptors, c-erb-B2, cytokeratin 7 and positive for cytocheratin 20 and CDX2 (Figures 3,4 and 5). Morphological and immunohistochemical findings supported the origin from colon cancer of neoplastic cells both in the breast lesion and the SNL micrometastasis.

## Conclusions

To our knowledge, this is the first case, in the English literature, showing findings consistent with metastasis to axillary SNL, related to a metastasis to the breast from a colon adenocarcinoma.

Breast metastases from colon cancer are very rare and they are usually associated with poor prognosis, due to disseminated disease. Since their first description, in 1976 [4], only 20 cases have been reported so far [5]. The majority of patients have undergone surgical excision even though breast surgery, in these cases, was aimed only at diagnosis and/or palliation. In the present case we opted for surgery, despite the presence of lung metastases, for two reasons. First, the diagnosis was not completely clear between a metastasis from colon cancer and a primary breast cancer. Second, the patient was very anxious about her breast lump and wanted it removed. She also asked for a sentinel node biopsy. Interestingly, the sentinel node turned out to harvest a tiny micrometastasis that makes this case rather unusual. Even though it is possible that micrometastasis could have been blood borne, it is more likely that colon cancer cells firstly deposited in the breast, and then, via the breast lymphatic vessels, reached the sentinel node. Accordingly, no disseminated nodal metastases were evident on CT and US before breast surgery.

The possible derivation of micrometastasis from the breast metastasis is in keeping with the general mechanism by which only a small proportion of disseminated cells become micrometastases and only a fraction of micrometastases progress to become macrometastases [2]. Moreover, the cancer cell invasion of breast peritumoral lymphatics should suggest that metastases (or even recurrences) from solid cancers are able to further metastasize, via well-known pathways also recognized in primary breast cancer [6].

## Authors' contributions

TP conceived of the study, participated in its design and drafted the manuscript.

VC participated in the design of the study and coordination, and drafted the manuscript.

LM participated in the design of the study.

SM participated in the design of the study and drafted the manuscript.

All authors read and approved the final manuscript.

## Consent

Written informed consent was obtained from the patient for publication of this case report and any accompanying images. A copy of the written consent is available for review by the Editor-in-Chief of this journal

## Competing interests

The authors declare that they have no competing interests.

## Funding

This work was supported in part by the research project "Integrazione delle attività di ricerca attraverso la costruzione di strutture e reti di collaborazione interistituzionali - Rete Nazionale Telepatologia (TESEO)" ACC/R8.5 - Roma - to VC; by the research project "Ruolo delle fosfoproteine nella chemioresistenza delle cellule staminali tumorali di colon e retto con analisi comparativa immunofenotipica", Grant from ISS - Roma: 527/B/3A/3 to VC.
